# Adjunctive treatment of brexpiprazole with fluoxetine shows a rapid antidepressant effect in social defeat stress model: Role of BDNF-TrkB signaling

**DOI:** 10.1038/srep39209

**Published:** 2016-12-19

**Authors:** Min Ma, Qian Ren, Chun Yang, Ji-chun Zhang, Wei Yao, Chao Dong, Yuta Ohgi, Takashi Futamura, Kenji Hashimoto

**Affiliations:** 1 Division of Clinical Neuroscience, Chiba University Center for Forensic Mental Health, Chiba, Japan; 2 Department of CNS Research, New Drug Research Division, Otsuka Pharmaceutical Co., Ltd., Tokushima, Japan

## Abstract

Addition of low doses of the atypical antipsychotic drug brexpiprazole with selective serotonin reuptake inhibitors (SSRIs) could promote antidepressant effect in patients with major depressive disorder although the precise mechanisms underlying the action of the combination are unknown. Combination of low dose of brexpiprazole (0.1 mg/kg) and SSRI fluoxetine (10 mg/kg) could promote a rapid antidepressant effect in social defeat stress model although brexpiprazole or fluoxetine alone did not show antidepressant effect. Furthermore, the combination significantly improved alterations in the brain-derived neurotrophic factor (BDNF) - TrkB signaling and dendritic spine density in the prefrontal cortex, hippocampus, and nucleus accumbens in the susceptible mice after social defeat stress. Interestingly, TrkB antagonist ANA-12 significantly blocked beneficial effects of combination of brexpiprazole and fluoxetine on depression-like phenotype. These results suggest that BDNF-TrkB signaling plays a role in the rapid antidepressant action of the combination of brexpiprazole and fluoxetine.

Substantial clinical data demonstrate that addition of low doses of atypical antipsychotic drugs (e.g., aripiprazole, olanzapine, quetiapine, risperidone, ziprasidone) to selective serotonin reuptake inhibitors (SSRIs) rapidly enhance the antidepressant effects in patients with major depressive disorder (MDD), including treatment-resistant patients^1^,^2^,^3^,^4^,^5^,^6^,^7^. Although clinical outcome of combined atypical antipsychotic drug and SSRI might be similar to ketamine’s induced rapid antidepressant effect^8^,^9^,^10^, the precise mechanisms underlying rapid antidepressant effect of the combination are currently unclear^11^,^12^.

Brexpiprazole (7-{4-[4-(1-benzothiophen-4-yl)piperazin-1-yl]butoxy}quinolin-2(1H)-one) is a serotonin-dopamine activity modulator^13^. Brexpiprazole binds with high affinity (Ki < 1 nM) to human serotonin (5-HT_1A_)-, 5-HT_2A_-, dopamine D_2_ (D_2L_)-, α_1B_-, and α_2C_-adrenergic receptors. It displays partial agonism at 5-HT_1A_ and D_2_ receptors, and potent antagonism of 5-HT_2A_ receptors and α_1B/2C_-adrenoceptors^13^. Furthermore, brexpiprazole was also shown to potentiate nerve growth factor (NGF)-induced neurite outgrowth in PC12 cells via 5-HT_1A_ and 5-HT_2A_ receptors^14^, suggesting that brexpiprazole may stimulate neuronal plasticity. Moreover, brexpiprazole showed antipsychotic-like and procognitive effects in rodents^15^,^16^,^17^. Brexpiprazole has been developed to offer efficacious and tolerable therapy for schizophrenia^18^,^19^,^20^,^21^,^22^. In addition, brexpiprazole was also developed as adjunctive therapy to antidepressants for the treatment of MDD^18^,^21^,^23^,^24^,^25^,^26^.

The purpose of this study is to examine whether brexpiprazole could demonstrate antidepressant-like effects in combination with sub-threshold dose of the SSRI fluoxetine in depression-like behaviors and alterations in the spine density in stress susceptible mice after repeated social defeat stress. It is well known that brain-derived neurotrophic factor (BDNF) and its receptor TrkB signaling plays a key role in the therapeutic mechanisms of the rapid antidepressants^27^,^28^,^29^,^30^,^31^,^32^,^33^. Therefore, we examined the role of BDNF-TrkB signaling in the mechanisms of a rapid antidepressant action of combination of brexpiprazole and fluoxetine.

## Results

### Effects of fluoxetine and brexpiprazole on depression-like behavior in susceptible mice after repeated social defeat stress

**Figure 1:**
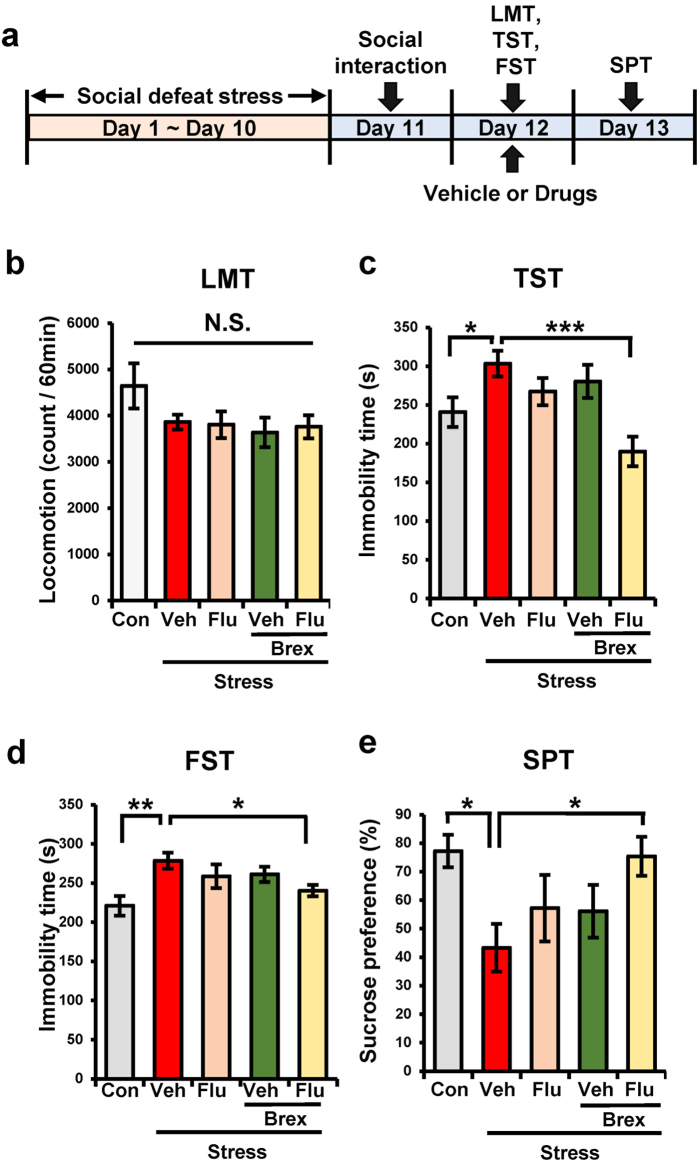
Antidepressant effects of combination of brexpiprazole and fluoxetine in social defeat stress model. (**a**): Schedule of social defeat stress, treatment, and behavioral tests. Repeated social defeat stress was performed 10 days (day 1- day 10). Social interaction test was performed day 11, and susceptible mice were used subsequent experiments. Vehicle (10 ml/kg), fluoxetine (10 mg/kg), brexpiprazole (0.1 mg/kg), or fluoxetine (10 mg/kg) plus brexpiprazole (0.1 mg/kg) were administered orally. Locomotion (LST), tail-suspension test (TST), and forced swimming test (FST) were performed 2, 4, and 6 hours after oral administration (day 12). One % sucrose preference test (SPT) was performed 24 hours after oral administration (day 13). (**b**): LMT, (**c**): TST, (**d**): FST, (**e**): SPT. Data are shown as mean ± S.E.M. (n = 6–9). *P < 0.05, **P < 0.01, ***P < 0.001 compared to vehicle-treated stress group (one-way ANOVA, followed post hoc LSD test). N.S.: Not significant.

We examined effects of fluoxetine and brexpiprazole on depression-like behavior after repeated social defeat stress. Vehicle, fluoxetine (10 mg/kg), brexpiprazole (0.1 mg/kg), or fluoxetine (10 mg/kg) plus brexpiprazole (0.1 mg/kg) was administered orally into susceptible mice (Fig. 1a). In the locomotion test (LMT), there were no differences (F_4,34_ = 1.347, P = 0.276) among the five groups (Fig. 1b). One-way ANOVA of TST and FST data revealed a significant result (TST: F_4,33_ = 6.139, P = 0.001, FST: F_4,43_ = 2.767, P = 0.043). In the TST and FST, combination of fluoxetine and brexpiprazole significantly reduced the increased immobility time in the susceptible mice after repeated social defeat stress (Fig. 1c,d). One-way ANOVA of SPT data revealed a significant result (F_4,38_ = 2.650, P = 0.048). In the SPT, combination of fluoxetine and brexpiprazole significantly increased the decreased sucrose preference of susceptible mice (Fig. 1e). In contrast, fluoxetine or brexpiprazole alone did not alter the immobility time for TST and FST, and decreased sucrose preference in the susceptible mice (Fig. 1c–e). These findings suggest that adjunctive treatment of brexpiprazole with fluoxetine showed a rapid antidepressant effect in the susceptible mice after repeated social defeat stress.

### Effects of fluoxetine and brexpiprazole on BDNF-TrkB signaling in selected brain regions of mice with depression-like phenotype

**Figure 2:**
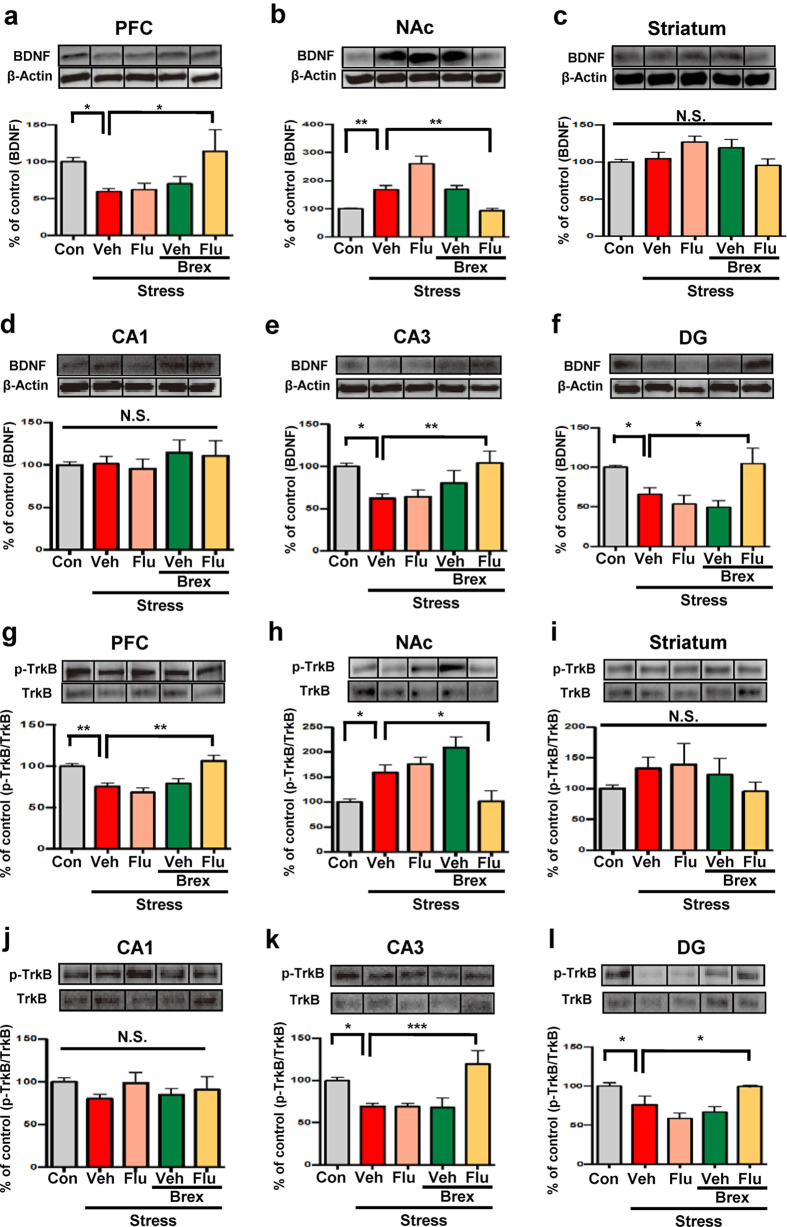
Effects of brexpiprazole and fluoxetine combination on the alterations in the BDNF-TrkB signaling in the brain regions from susceptible mice after social defeat stress. (**a**–**f**): Forty eight hours after administration of drugs, brain regions from mice were collected. Western blot analysis of BDNF (mature form) and β-actin in the brain regions (PFC, NAc, striatum, CA1, CA3, DG) was performed. The values are expressed as a percentage of that of control mice. Representative data of Western blot analyses of BDNF and β-actin in the mouse brain regions. Data are shown as mean ± S.E.M. (n = 5–8). *P < 0.05, **P < 0.01, ***P < 0.001 compared to vehicle-treated stress group (one-way ANOVA, followed post hoc LSD test). N.S.: Not significant. (**g**–**l**): The ratio of p-TrkB to total TrkB in the brain regions is shown. The values are expressed as a percentage of that of control mice. Representative data of Western blot analyses of p-TrkB, and TrkB in the mouse brain regions. Data are shown as mean ± S.E.M. (n = 5–8). *P < 0.05, **P < 0.01, ***P < 0.001 compared to vehicle-treated stress group (one-way ANOVA, followed post hoc LSD test). N.S.: Not significant.

Since PFC, NAc, striatum, CA1, CA3 and dentate gyrus (DG) of the hippocampus play a role in the depression-like phenotype in rodents^34^,^35^,^36^,^37^,^38^,^39^, we performed Western blot analysis of BDNF (mature form), its precursor proBDNF, TrkB and phosphorylated TrkB (p-TrkB) in selected brain regions (PFC, NAc, striatum, DG, CA1 and CA3). One-way ANOVA of BDNF data revealed the following statistical significances: PFC: F_4,27_ = 3.705, P = 0.016, NAc: F_4,27_ = 18.79, P < 0.0001, striatum: F_4,27_ = 1.934, P = 0.132, CA1: F_4,27_ = 0.381, P = 0.82; CA3: F_4,27_ = 4.227, P = 0.009; DG: F_4,27_ = 5.53, P = 0.002 (Fig. 2a–f). Combination of brexpiprazole and fluoxetine significantly attenuated decreased BDNF levels in the PFC, CA3 and DG regions, but not CA1 region, of susceptible mice (Fig. 2a,d–f). Interestingly, combination of brexpiprazole and fluoxetine significantly attenuated increased BDNF levels in the NAc of susceptible mice (Fig. 2b). However, protein levels of proBDNF in all the regions examined were not different among the five treatment groups (Supplemental Fig. 1a–f).

To clarify whether TrkB activation or inhibition is the underlying mechanism of action of brexpiprazole and fluoxetine combination, we performed Western blot analyses of TrkB and phosphorylated TrkB (p-TrkB), an activated form of TrkB, in samples from PFC, NAc, striatum, and CA1, CA3, DG of hippocampus. One-way ANOVA of p-TrkB/TrkB data revealed the following statistical results: PFC: F_4,27_ = 11.14, P < 0.0001, NAc: F_4,27_ = 6.095, P = 0.001, striatum: F_4,27_ = 0.698, P = 0.6, CA1: F_4,27_ = 0.764, P = 0.558; CA3: F_4,27_ = 6.149, P = 0.001; DG: F_4,27_ = 8.26, P = 0.0002 (Fig. 2g–l). Combination of brexpiprazole and fluoxetine significantly attenuated the decrease in p-TrkB/TrkB ratio in the PFC, CA3 and DG regions, but not CA1 region, of susceptible mice (Fig. 2g,j,k,l). Interestingly, combination of brexpiprazole and fluoxetine significantly attenuated the increase of p-TrkB/TrkB ratio in the NAc of susceptible mice (Fig. 2h). However, protein levels of TrkB in all these regions were not different among the five groups (Supplemental Fig. 1g–l).

### Effects of combination of fluoxetine and brexpiprazole on dendritic spines density in selected brain regions of mice with depression-like phenotype

**Figure 3:**
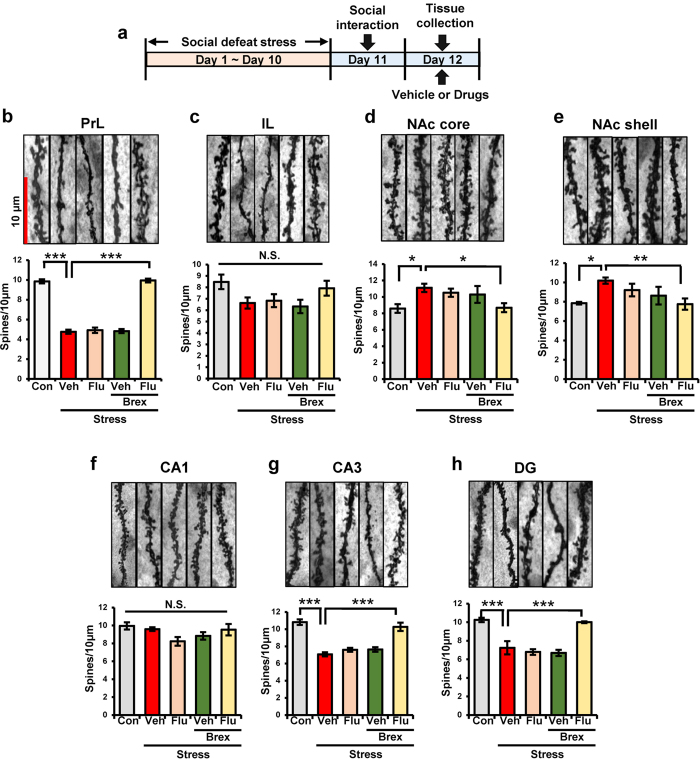
Effects of brexpiprazole and fluoxetine combination on the alterations in the dendritic spine density in the brain regions from susceptible mice after social defeat stress. (**a**): Schedule of social defeat stress, treatment, and behavioral tests. Repeated social defeat stress was performed 10 days (day 1- day 10). Social interaction test was performed day 11, and susceptible mice were used subsequent experiments. Two hours after oral administration of vehicle (10 ml/kg), fluoxetine (10 mg/kg), brexpiprazole (0.1 mg/kg), or fluoxetine (10 mg/kg) plus brexpiprazole (0.1 mg/kg), brains from mice were collected for Golgi staining. (**b**–**h**): Golgi staining in the brain regions (PrL and IL regions of mPFC, core and shell of NAc, CA1, CA3, DG of hippocampus) was performed. Representative data of Golgi staining in the mouse brain regions. Data are shown as mean ± S.E.M. (n = 5 or 6). *P < 0.05, **P < 0.01, ***P < 0.001 compared to vehicle-treated stress group (one-way ANOVA, followed post hoc LSD test). N.S.: Not significant.

Repeated social defeat stress causes alterations in the dendritic spines density in the PFC, CA3 and DG of hippocampus, and NAc^37^,^39^. In this study, we examined whether combination of brexpiprazole and fluoxetine could cause alterations in the dendritic spines density in the prelimbic (PrL) and infralimbic (IL) regions of mPFC, shell and core of NAc, CA1, CA3 and DG of the hippocampus (Fig. 3a–h). One-way ANOVA of Golgi staining data revealed the following statistical results: (PrL of mPFC: F_4,21_ = 158.0, P < 0.0001, IL of mPFC: F_4,20_ = 2.44, P = 0.08, NAc core: F_4,20_ = 3.305, P = 0.042, NAc shell: F_4,20_ = 2.96, P = 0.045, CA1: F_4,21_ = 2.294, P = 0.093; CA3: F_4,21_ = 28.79, P < 0.0001; DG: F_4,21_ = 20.51, P < 0.0001)(Fig. 3b–i). Combination of brexpiprazole and fluoxetine significantly attenuated decreased spine density in the PrL of mPFC, CA3 and DG regions of susceptible mice (Fig. 3b,g,h). Interestingly, combination of brexpiprazole and fluoxetine significantly attenuated the increase in spine density in the core and shell of NAc of susceptible mice (Fig. 3d,e). In contrast, administration of brexpiprazole or fluoxetine alone did not affect the alterations in the dendritic spines density in these regions (Fig. 3b–h).

### Role of TrkB in the mechanism of action of brexpiprazole and fluoxetine combination in the social defeat stress model

**Figure 4:**
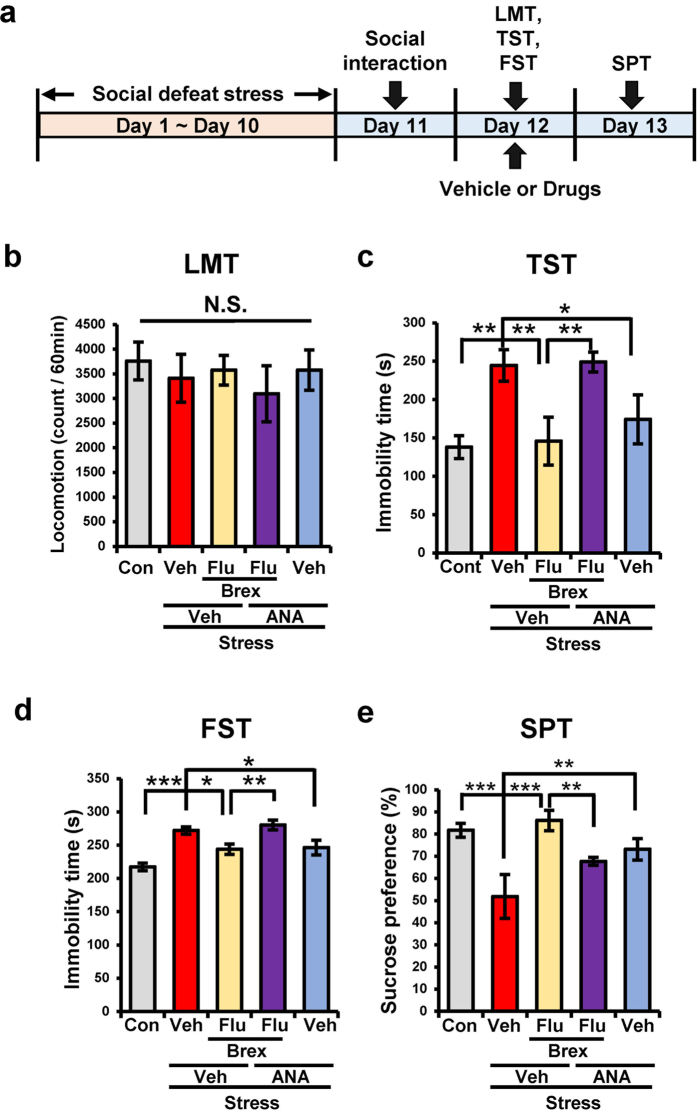
Effects of TrkB antagonist ANA-12 on antidepressant effect of combination of brexpiprazole and fluoxetine. (**a**): Schedule of social defeat stress, treatment, and behavioral tests. Repeated social defeat stress was performed 10 days (day 1- day 10). Social interaction test was performed day 11, and susceptible mice were used subsequent experiments. Vehicle (10 ml/kg), fluoxetine (10 mg/kg), brexpiprazole (0.1 mg/kg), or fluoxetine (10 mg/kg) plus brexpiprazole (0.1 mg/kg) were administered orally 30 min after administration of vehicle or ANA-12 (0.5 mg/kg). Locomotion (LST), tail-suspension test (TST), and forced swimming test (FST) were performed 2, 4, and 6 hours after oral administration (day 12). One % sucrose preference test (SPT) was performed 24 hours after oral administration (day 13). (**b**): LMT, (**c**): TST, (**d**): FST, (**e**): SPT. Data are shown as mean ± S.E.M. (n = 6–9). *P < 0.05, **P < 0.01, ***P < 0.001 compared to vehicle-treated stress group (one-way ANOVA, followed post hoc LSD test). N.S.: Not significant.

To assess the role of TrkB on the mechanism of action of combination of brexpiprazole and fluoxetine, we examined the effects of ANA-12, a novel TrkB antagonist^40^, on depression-like behavior after social defeat stress (Fig. 4a). One-way ANOVA of the behavioral data showed the following statistical results: (LMT: F_4,35_ = 0.287, P = 0.884, TST: F_4,34_ = 6.163, P = 0.0008, FST: F_4,33_ = 12.73, P < 0.0001, SPT: F_4,33_ = 9.022, P < 0.0001) (Fig. 4b–e). In the TST and FST, treatment with ANA-12 significantly blocked the effects of brexpiprazole plus fluoxetine on the increased immobility time of susceptible mice (Fig. 4c,d). Furthermore, treatment with ANA-12 significantly blocked the effects of brexpiprazole plus fluoxetine on the decreased sucrose preference of susceptible mice (Fig. 4e). In addition, ANA-12 alone showed antidepressant effects in these tests, consistent with previous reports^37^,^38^,^39^.

### Role of BDNF-TrkB signaling in the mechanism of action of brexpiprazole and fluoxetine combination

**Figure 5:**
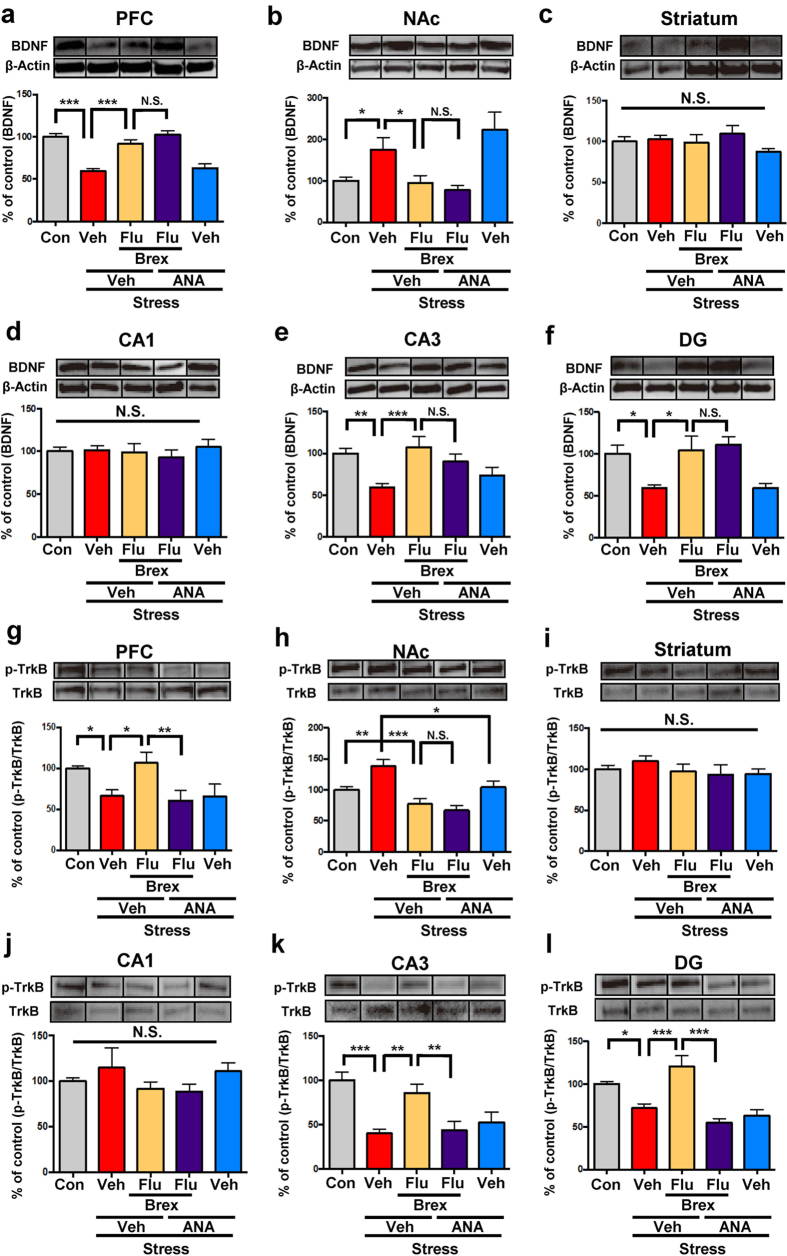
Effect of TrkB antagonist ANA-12 on the alterations in the BDNF-TrkB signaling in the brain regions from susceptible mice after social defeat stress. (**a**–**f**): Forty eight hours after administration of drugs, brain regions from mice were collected. Western blot analysis of BDNF (mature form) and β-actin in the brain regions (PFC, NAc, striatum, CA1, CA3, DG) was performed. The values are expressed as a percentage of that of control mice. Data are shown as mean ± S.E.M. (n = 6–8). *P < 0.05, **P < 0.01, ***P < 0.001 compared to vehicle-treated stress group (one-way ANOVA, followed post hoc LSD test). N.S.: Not significant. (**g**–**l**): The ratio of p-TrkB to total TrkB in the brain regions is shown. Representative data of Western blot analyses of p-TrkB, and TrkB in the mouse brain regions. The values are expressed as a percentage of that of control mice. Data are shown as mean ± S.E.M. (n = 6–8). *P < 0.05, **P < 0.01, ***P < 0.001 compared to vehicle-treated stress group (one-way ANOVA, followed post hoc LSD test). N.S.: Not significant.

Since TrkB antagonist ANA-12 blocked antidepressant effect of brexpiprazole and fluoxetine combination in the social defeat stress model, we performed Western blot analysis of BDNF-TrkB signaling in the brain regions. One-way ANOVA of BDNF data showed the following statistical results: (PFC: F_4,28_ = 25.33, P < 0.0001, NAc: F_4,28_ = 7.179, P = 0.001, striatum: F_4,28_ = 1.203, P = 6.331, CA1: F_4,28_ = 0.349, P = 0.843, CA3: F_4,28_ = 5.532, P = 0.002, DG: F_4,29_ = 4.112, P = 0.009) (Fig. 5a–f). Combination of brexpiprazole and fluoxetine significantly attenuated the decrease in levels of BDNF in the PFC, CA3, and DG from susceptible mice after social defeat stress (Fig. 5a,e,f). However, treatment with ANA-12 did not alter the levels of BDNF in these brain regions.

One-way ANOVA of p-TrkB/TrkB ratio data showed the following statistical results: (PFC: F_4,28_ = 4.642, P = 0.005, NAc: F_4,29_ = 7.305, P < 0.0003, striatum: F_4,28_ = 0.545, P = 0.704, CA1: F_4,28_ = 1.063, P = 0.393, CA3: F_4,29_ =7.056, P = 0.0001, DG: F_4,29_ = 9.935, P < 0.0001) (Fig. 5g–l). Combination of brexpiprazole and fluoxetine significantly attenuated the decreased p-TrkB/TrkB ratio in the PFC, CA3, and DG from susceptible mice after social defeat stress (Fig. 5g,k,l). Furthermore, treatment with ANA-12 significantly blocked the effect of the combination of brexpiprazole and fluoxetine in these regions (PFC, CA3, DG), suggesting a role of TrkB in the mechanism of action of the combination therapy. Although combination of brexpiprazole and fluoxetine significantly attenuated the increased p-TrkB/TrkB ratio in the NAc of susceptible mice, ANA-12 did not block the effect of the combination (Fig. 5h). However, treatment with ANA-12 alone significantly attenuated the increase of p-TrkB/TrkB ratio in the NAc of susceptible mice, consistent with previous reports^37^,^38^,^39^. Protein levels of proBDNF and TrkB in the all brain regions were not significantly different among the five groups (Supplemental Fig. 2a–l).

## Discussion

The key findings of this study demonstrate that although brexpipazole or fluoxetine alone did not show antidepressant effect, their combination could promote a rapid antidepressant effect in the social defeat stress model of depression. Recently, we reported a rapid antidepressant effect of the *N*-methyl-D-aspartate (NMDA) receptor antagonist ketamine (or R-ketamine) in the same model^37^,^39^,^41^, indicating that the rapid antidepressant effect of combination of brexpiprazole and fluoxetine might be similar to ketamine’s rapid antidepressant action. A recent study demonstrated that, similar to ketamine, a combination of olanzapine and fluoxetine facilitated NMDA- and AMPA-induced currents in pyramidal cells via activation of dopamine D_1_ receptors^42^, suggesting that rapid antidepressant effect of both antipsychotic drug and SSRI may be related to a common mechanism of action. To the best of our knowledge, this is the first report showing a rapid antidepressant effect for brexpiprazole plus fluoxetine in the social defeat stress model. Therefore, adjunctive therapy of brexpiprazole with SSRI could promote a rapid antidepressant effect in MDD patients. Interestingly, we showed that TrkB antagonist ANA-12 significantly blocked the rapid antidepressant effect of combination of brexpiprazole and fluoxetine in this model. Given the role of BDNF-TrkB signaling in the antidepressant effects of ketamine^37^,^43^,^44^,^45^,^46^, it is therefore likely that BDNF-TrkB signaling may also play a key role in the rapid antidepressant effect of combination of brexpiprazole and fluoxetine in the social defeat stress model.

We previously reported a marked reduction of BDNF-TrkB signaling in the PFC, DG and CA3, but not CA1, of inflammation model^38^, repeated social defeat stress model^37^,^39^,^41^ and learned helplessness model^35^,^36^,^47^. Direct infusion of BDNF (or TrkB agonist 7,8-dihydroxyflavone (7,8-DHF)^48^) into the DG and CA3, but not CA1, promoted rapid and sustained antidepressant effects in the rat learned helplessness model of depression^35^,^49^, thus implicating the BDNF-TrkB signal pathway in the DG, and CA3, but not CA1, in the antidepressant action of BDNF or TrkB agonist. This is consistent with decreased BDNF protein levels seen in the PFC, DG, CA3, but not CA1, in rat learned helplessness model^35^,^36^. In the present study, we found that combination of brexpiprazole and fluoxetine could attenuate decreased BDNF-TrkB signaling in the PFC, CA3, and DG from susceptible mice after social defeat stress. We also reported that 7,8-DHF promoted a rapid antidepressant effect in social defeat stress model^39^. Therefore, it is likely that combination of brexpiprazole and fluoxetine might promote a rapid antidepressant effect by stimulating BDNF-TrkB pathway in these regions.

The ventral tegmental area (VTA)-NAc pathway plays a critical role in the depression-phenotype^35^,^36^,^38^,^39^,^41^,^50^. We reported that inflammation, social defeat stress and learned helplessness caused an increased BDNF-TrkB signaling within the NAc^35^,^36^,^37^,^38^,^39^. Thus, social defeat stress causes decreased BDNF-TrkB signaling in the hippocampus and PFC, but an increased BDNF-TrkB signaling in the NAc, resulting in depression-like behavior in mice. Interestingly, we found that combination of brexpiprazole and fluoxetine could attenuate the alterations in the BDNF-TrkB signaling in the PFC, hippocampus as well as NAc. In contrast, we also found that ketamine did not alter the increased levels of BDNF in the NAc from susceptible mice after social defeat stress^37^,^39^, suggesting that ketamine can induce the rapid and long-lasting antidepressant effects by increasing BDNF in the PFC and hippocampus, but not in NAc. It is noteworthy that combination of brexpiprazole and fluoxetine could improve alterations in the BDNF-TrkB signaling in the NAc from susceptible mice after social defeat stress.

Changes in dendritic length and spines density in the PFC and hippocampus are thought to contribute to the neurobiology of depression, and antidepressant treatment is mediated, in part, by blocking or reversing these changes^51^,^52^,^53^. Recently, we reported that ketamine or R-ketamine showed a rapid antidepressant activity by normalizing altered dendritic spines in the PFC and hippocampus, but not NAc^37^,^39^. In addition, we also reported that ketamine did not show antidepressant effect in depression-like behavior induced by increased BDNF-TrkB signaling in the NAc after methamphetamine withdrawal^34^). Together, it is likely that NAc may not be involved in the antidepressant effect of ketamine. A single administration of 7,8-DHF or ANA-12 could normalize alterations in spines density in the social defeat stress model by stimulation at TrkB in the PFC, CA3, and DG, as well as blockade of TrkB in the NAc^54^. Therefore, it is likely that combination of brexpiprazole and fluoxetine could act by normalizing altered dendritic spines density in regions such as PFC, hippocampus, and NAc. Accordingly, it is likely that, contrary to ketamine, BDNF-TrkB signaling in NAc might be necessary to mediate the antidepressant effect of the combination brexpiprazole plus fluoxetine, although further studies are needed.

In conclusion, this study shows that adjunction of brexpiprazole to fluoxetine can produce a rapid antidepressant effect in the social defeat stress model of depression and that BDNF-TrkB signaling plays a role in the rapid antidepressant action of such combination therapy. Therefore, it is likely that adjunction of brexpiprazole to SSRI could produce a rapid antidepressant effect in treatment-resistant patients with MDD, but without the ketamine-induced psychotomimetic effects and abuse potential.

## Methods and Materials

### Animals

Male adult C57BL/6 mice 8 weeks old weighing 20–25 g and male adult CD1 mice aged 13–15 weeks (body weight >40 g) were purchased from SLC Japan (Hamamatsu, Shizuoka, Japan). The mice were housed in clear polycarbonate cages (22.5 × 33.8 × 14.0 cm) in groups of 4 or 5 individuals under a controlled 12/12-h light–dark cycle (light from 7:00 AM to 7:00 PM), with the room temperature kept at 23 ± 1 °C and humidity at 55 ± 5%. The mice were given free access to water and food pellets specifically designed for mice. All experiments were carried out in accordance with the Guide for Animal Experimentation of Chiba University. The protocol was approved by the Chiba University Institutional Animal Care and Use Committee.

### Drugs and drug administration

Brexpiprazole was synthesized at Otsuka Pharmaceutical Co., Ltd. (Tokyo, Japan). Vehicle (0.5% CMC; 10 ml/kg), fluoxetine (10 mg/kg, Wako Chemical Co., Ltd, Tokyo, Japan), brexpiprazole (0.1 mg/kg), or fluoxetine (10 mg/kg) plus brexpiprazole (0.1 mg/kg) were administered orally into mice. To study the role of BDNF-TrkB signaling in the mechanism of fluoxetine plus brexpiprazole, vehicle (17% dimethyl sulfoxide (DMSO) in phosphate-buffered saline) or ANA-12 (N-[2-[[(Hexahydro-2-oxo-1H-azepin-3-yl) amino] carbonyl] phenyl]-benzo[b]thiophene-2-carboxamide; 0.5 mg/kg, Maybridge, Loughborough, Leicestershire, UK)) was administrated intraperitoneally (i.p.) into mice 30 min before drug administration. The doses of brexpiprazole (0.1 mg/kg), fluoxetine (10 mg/kg) and ANA-12 (0.5 mg/kg) were selected as reported previously^11^,^13^,^15^,^16^,^17^,^38^,^39^,^40^,^41^. Other chemicals were purchased from commercial sources.

### Social defeat procedure

The procedure of social defeat stress was performed as previously reported^37^,^39^,^41^,^54^,^55^,^56^,^57^. Every day the C57BL/6 mice were exposed to a different CD1 aggressor mouse for 10 min, total for 10 days. When the social defeat session ended, the resident CD1 mouse and the intruder mouse were housed in one half of the cage separated by a perforated Plexiglas divider to allow visual, olfactory and auditory contact for the remainder of the 24- h period. At 24 h after the last session, all mice were housed individually. On day 11, a social avoidance test was performed to identify subgroups of mice that were susceptible and unsusceptible to social defeat stress. This was accomplished by placing mice in an interaction test box (42 × 42 cm) with an empty wire-mesh cage (10 × 4.5 cm) located at one end. The movement of the mice was tracked for 2.5 min, followed by 2.5 min in the presence of an unfamiliar aggressor confined in the wire-mesh cage. The duration of the subject’s presence in the ‘interaction zone’ (defined as the 8-cm-wide area surrounding the wire-mesh cage) was recorded by a stopwatch. The interaction ratio was calculated as the time spent in the interaction zone with an aggressor/time spent in the interaction zone without an aggressor. An interaction ratio of 1 was set as the cutoff: mice with scores <1 were defined as ‘susceptible’ to social defeat stress and those with scores ≥1 were defined as ‘unsusceptible’. Only susceptible mice were used in the subsequent experiments.

### Behavioral tests of antidepressant effects

Behavioral tests were performed as previously reported^37^,^39^,^41^,^54^,^55^,^56^,^57^.

#### Locomotion (LMT)

Mice were placed in experimental cages (L560 × W560 × H330 mm), and locomotor activity was counted by the SCANET MV-40 (MELQUEST, Toyama, Japan). The cumulative locomotor activity was recorded for 60 min. All cages were cleaned between test sessions.

#### Tail suspension test (TST)

A small piece of adhesive tape was afixed 2 cm from the tip of the tail and punched with a single hole through which mice were hung individually, on a hook. The immobility time of each mouse was recorded for 10 min. Mice were considered immobile only when they hung passively and completely motionless. The TST were performed 2 h after the LMT.

#### Forced swimming test (FST)

Animals were tested in an automated forced-swim apparatus using SCANET MV-40 (MELQUEST Co., Ltd., Toyama, Japan). The mice were placed individually in a cylinder (Diameter 23 cm; Height 21 cm), containing 15 cm of 23 ± 1 °C warm water. Immobility time was calculated by subtracting active time from total time, using the apparatus analysis software. Cumulative immobility time was scored for 6 min during the test. The TST and FST were performed 2 and 4 h after the LMT, respectively.

#### Sucrose preference test (SPT)

Mice were exposed to water and 1% sucrose solution for 48 h, followed by 4 h of water and food deprivation and a 1 h exposure to two identical bottles, one containing water and the other 1% sucrose solution. These bottles were weighed before and at the end of the 1 h test period and the sucrose preference (%) was determined.

### Western blot analysis of BDNF, and its precursor proBDNF, TrkB, and phosphorylated-TrkB

Western blot analysis was performed as reported previously^37^,^39^,^41^,^54^,^55^,^56^,^57^. Mice were killed by cervical dislocation and brains were rapidly removed from the skull. Approximately 1-mm-thick coronal sections were cut and bilateral tissue punches of prefrontal cortex (PFC), nucleus accumbens (NAc), striatum, CA1, CA3, and dentate gyrus (DG) of the hippocampus were dissected on ice using a SZ-LED Kenis light microscope (Osaka, Japan), and stored at −80 °C. Basically, tissue samples were homogenized in Laemmli lysis buffer. Aliquots (20 μg) of protein were measured using the DC protein assay kit (Bio-Rad), and incubated for 5 min at 95 °C, with an equal volume of 125 mM Tris-HCl, pH 6.8, 20% glycerol, 0.1% bromophenol blue, 10% β-mercaptoethanol, 4% SDS, and subjected to SDS polyacrylamide gel electrophoresis using AnyKD minigels (Mini-PROTEAN TGX Precast Gel; BioRad). Proteins were transferred onto PVDF membranes using a Trans Blot Mini Cell (Bio-Rad). For immunodetection, the blots were blocked with 2% BSA in TBST (TBS + 0.1% Tween-20) for 1 h at room temperature, and kept with primary antibodies overnight at 4 °C. The following primary antibodies were used: BDNF (1:200; H-117, Cat#: sc-20981, Santa Cruz Biotechnology), phosphor-TrkB (Tyr-706) (1:200; Cat#: sc135645, Santa Cruz Biotechnology), TrkB (80E3) (1:1,000; Cat#: 4603, Cell Signaling Technology). The next day, blots were washed three times in TBST, and incubated with horseradish peroxidase-conjugated anti-rabbit antibody (1:10,000) 1 hour at room temperature. After a final three washes with TBST, bands were detected using enhanced chemiluminescence (ECL) plus the Western Blotting Detection system (GE Healthcare Bioscience). The blots then were washed three times in TBST and incubated with the primary antibody directed against β-actin (1:10,000; Sigma-Aldrich). Images were captured with a Fuji LAS3000-mini imaging system (Fujifilm, Tokyo, Japan), and immunoreactive bands were quantified.

### Golgi Staining

Golgi staining was performed using the FD Rapid GolgiStain^TM^ Kit (FD Neuro Technologies, Inc., Columbia, MD), following the manufacturer’s instructions^34^,^38^. Twenty four hours after oral administration of vehicle (10 ml/kg), fluoxetine (10 mg/kg), brexpiprazole (0.1 mg/kg), or fluoxetine (10 mg/kg) plus brexpiprazole (0.1 mg/kg), animals were deeply anesthetized with sodium pentobarbital, and brains were removed from the skull and rinsed in double distilled water. Brains were immersed in the impregnation solution, made by mixing equal volumes of Solution A and B, overnight and then stored in fresh solution, for 2 weeks in the dark. Brains were transferred into Solution C overnight and then stored in fresh solution at 4 °C for 1 week, in the dark. Coronal brain sections (100 μm thickness) were cut on a cryostat (3050 S, Leica Microsystems AG, Wetzlar, Germany), with the chamber temperature set at −20 °C. Each section was mounted in Solution C, on saline-coated microscope slides. After absorption of excess solution, sections were dried naturally, at room temperature. Dried sections were processed following the manufacturer’s instructions. Briefly, images of dendrites within CA1, CA3, and DG of the hippocampus, prelimbic (PrL) and inflalimbic (IL) areas of medial PFC (mPFC), and shell and core of NAc were captured using a 100x objective with a Keyence BZ-9000 Generation II microscope (Osaka, Japan). Spines were counted along CA1, CA3, DG, PrL and IL of mPFC and NAc dendrites starting from their point of origin from the primary dendrite, as previously reported^34^,^38^. For spine density measurements, all clearly evaluable areas containing 50–100 μm of secondary dendrites from each imaged neuron were used. To determine relative spine density, spines on multiple dendritic branches from a single neuron were counted to obtain an average spine number per 10 μm. For spine number measurements, only spines that emerged perpendicular to the dendritic shaft were counted. Three neurons per section, three sections per animal and six animals were analyzed. The average value for each region, in each individual was obtained. These individual averages were then combined to yield a grand average for each region.

### Statistical Analysis

The data show as the mean ± standard error of the mean (S.E.M.). Analysis was performed using PASW Statistics 20 (formerly SPSS Statistics; SPSS). Comparisons between groups were performed using the one-way analysis of variance (ANOVA), followed by post hoc Fisher’s Least Significant Difference (LSD) tests. The P values of less than 0.05 were considered statistically significant.

## Additional Information

**How to cite this article**: Ma, M. *et al*. Adjunctive treatment of brexpiprazole with fluoxetine shows a rapid antidepressant effect in social defeat stress model: Role of BDNF-TrkB signaling. *Sci. Rep.* **6**, 39209; doi: 10.1038/srep39209 (2016).

**Publisher's note:** Springer Nature remains neutral with regard to jurisdictional claims in published maps and institutional affiliations.

## Supplementary Material

Supplementary Information
