# Supplementary material for: Adjunctive treatment of brexpiprazole with fluoxetine shows a rapid antidepressant effect in social defeat stress model: Role of BDNF-TrkB signaling
Source: Sci Rep. 2016 Dec 19;6:39209. doi: 10.1038/srep39209 (PMC5171769; doi:10.1038/srep39209)

Supplemental information

Adjunctive treatment of brexpiprazole with fluoxetine shows a rapid antidepressant effect in social defeat stress model: Role of BDNF-TrkB signaling

Min Ma[a], Qian Ren[a], Chun Yang[a], Ji-chun Zhang[a], Wei Yao[a], Chao Dong[a], Yuta Ohgi[b], Takashi Futamura[b] and Kenji Hashimoto[a,*]

[a]Division of Clinical Neuroscience, Chiba University Center for Forensic Mental Health, Chiba, Japan, and [b]Department of CNS Research, New Drug Research Division, Otsuka Pharmaceutical Co., Ltd., Tokushima, Japan

Correspondence and requests for materials should be addressed to Dr. Kenji Hashimoto, Division of Clinical Neuroscience, Chiba University Center for Forensic Mental Health, Inohana 1-8-1, Chiba, 260-8670, Japan; TEL: +81-43-226-2517, FAX: +81-43-226-2561
E-mail: hashimoto@faculty.chiba-u.jp

Supplemental Figure 1. Effects of brexpiprazole and fluoxetine on the levels of proBDNF and TrkB in the brain regions

(a-f): Forty eight hours after administration of drugs, brain regions from mice were collected. Western blot analysis of proBDNF and β-actin in the brain regions (PFC, NAc, striatum, CA1, CA3, DG) was performed. The values are expressed as a percentage of that of control mice. Representative data of Western blot analyses of proBDNF and β-actin in the mouse brain regions. Data are shown as mean ± S.E.M. (n = 5 - 8). N.S.: Not significant. (g-l): Western blot analysis of TrkB and β-actin in the brain regions (PFC, NAc, striatum, CA1, CA3, DG) was performed. Representative data of Western blot analyses of TrkB, and β-actin in the mouse brain regions. The values are expressed as a percentage of that of control mice. Data are shown as mean ± S.E.M. (n = 5 - 8). N.S.: Not significant.

Supplemental Figure 2. Effects of ANA-12 on the levels of proBDNF and TrkB in the brain regions

(a-f): Forty eight hours after administration of drugs, brain regions from mice were collected. Western blot analysis of proBDNF and β-actin in the brain regions (PFC, NAc, striatum, CA1, CA3, DG) was performed. The values are expressed as a percentage of that of control mice. Representative data of Western blot analyses of proBDNF and β-actin in the mouse brain regions. Data are shown as mean ± S.E.M. (n = 6 - 8). N.S.: Not significant. (g-l): Western blot analysis of TrkB and β-actin in the brain regions (PFC, NAc, striatum, CA1, CA3, DG) was performed. Representative data of Western blot analyses of TrkB, and β-actin in the mouse brain regions. The values are expressed as a percentage of that of control mice. Data are shown as mean ± S.E.M. (n = 6 - 8). N.S.: Not significant.

Supplemental Figure 3. Western blot of proteins (p-TrkB, TrkB, proBDNF, BDNF, beta-actin) in the brain regions

(a): PFC, (b): Nac, (c): Striatum, (d): CA1, (e): CA3, (f): DG
The red line marked blots were selected in the Figure 2.

Supplemental Figure 4. Western blot of proteins (p-TrkB, TrkB, proBDNF, BDNF, beta-actin) in the brain regions

(a): PFC, (b): Nac, (c): Striatum, (d): CA1, (e): CA3, (f): DG
The red line marked blots were selected in the Figure 5.

Supplemental Figure 1

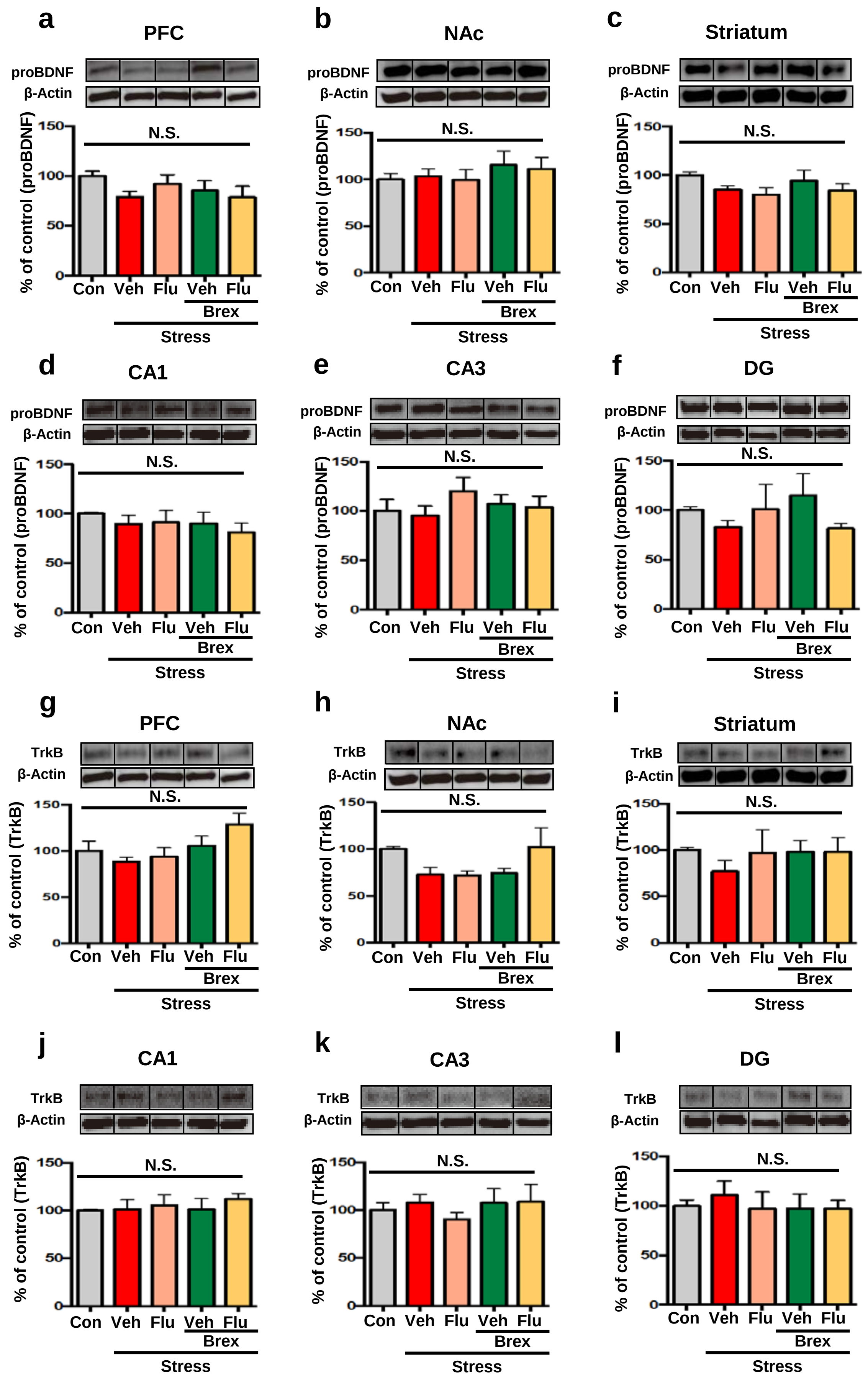

Supplemental Figure 2

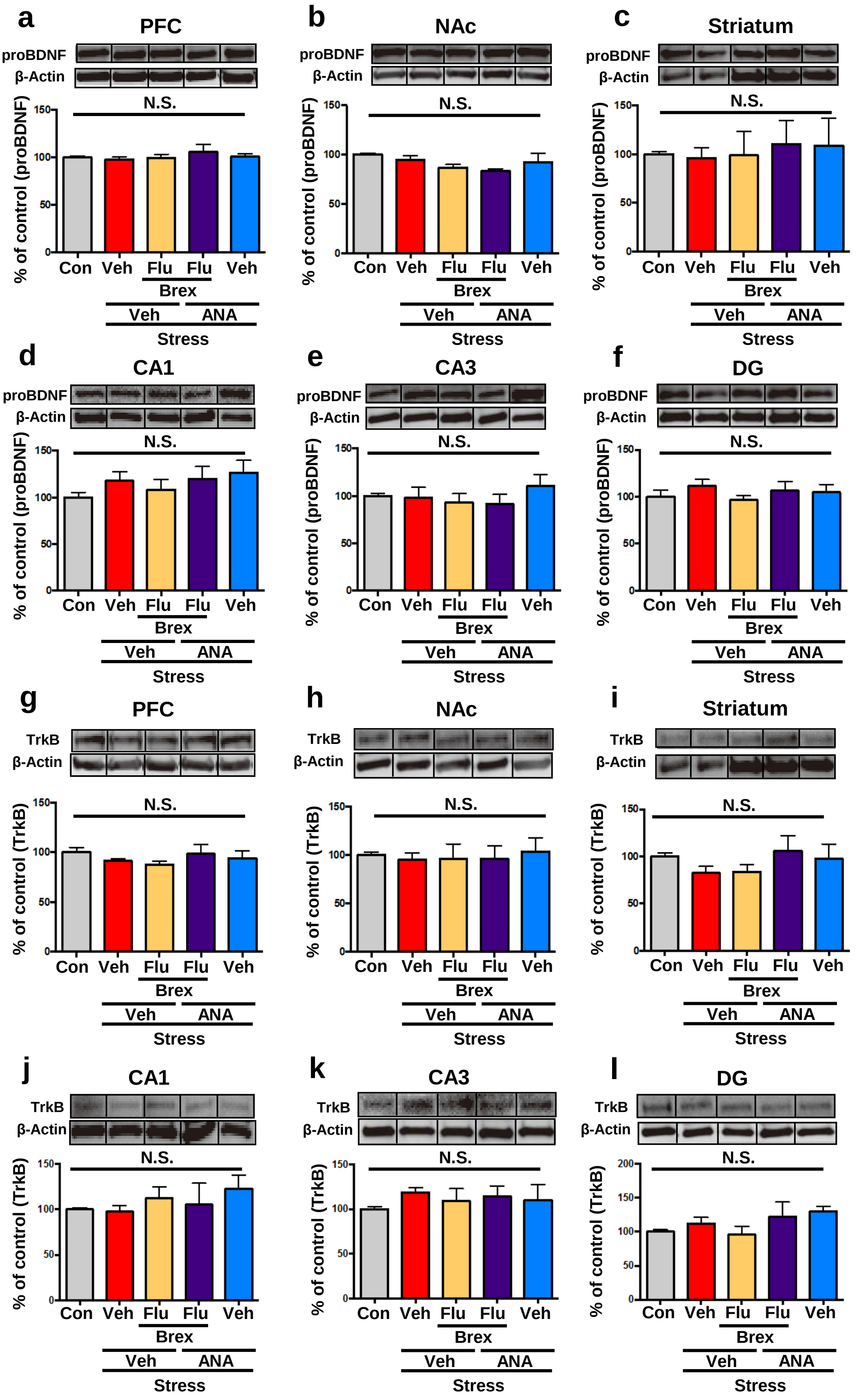

Supplemental Figure 3

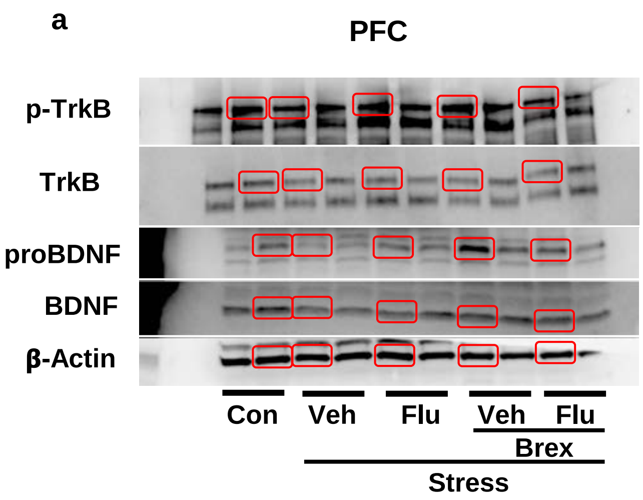

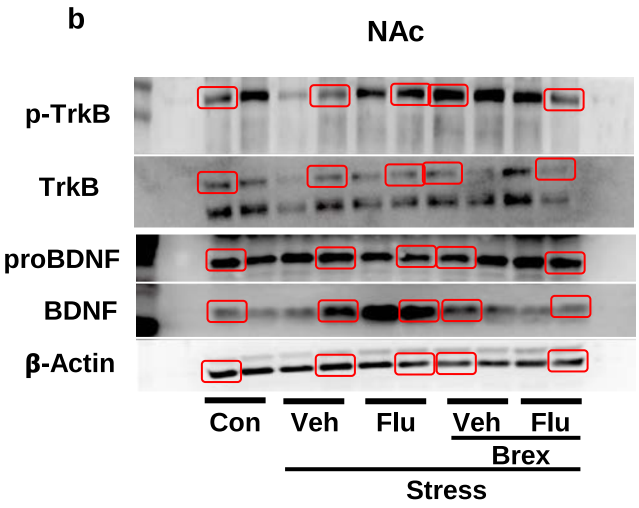

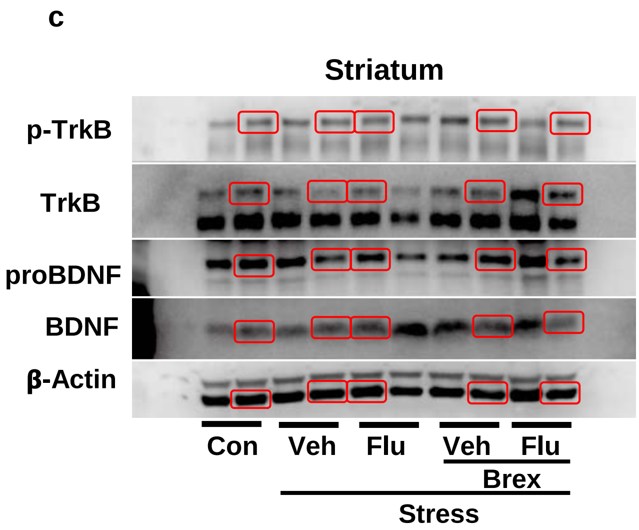

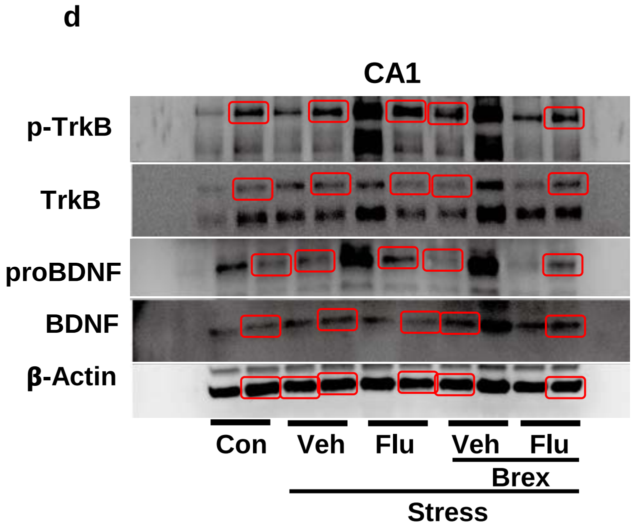

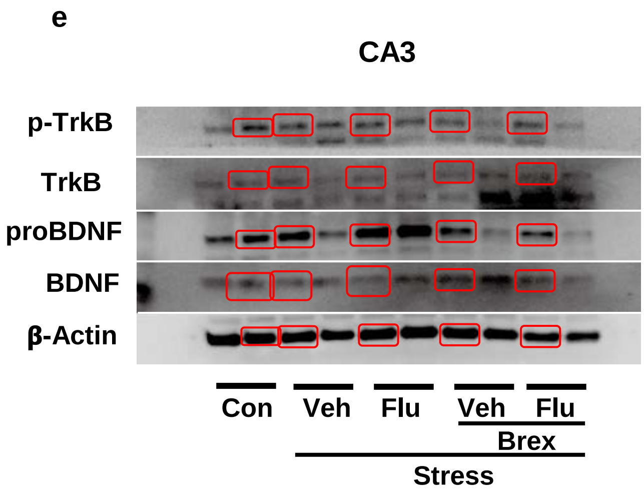

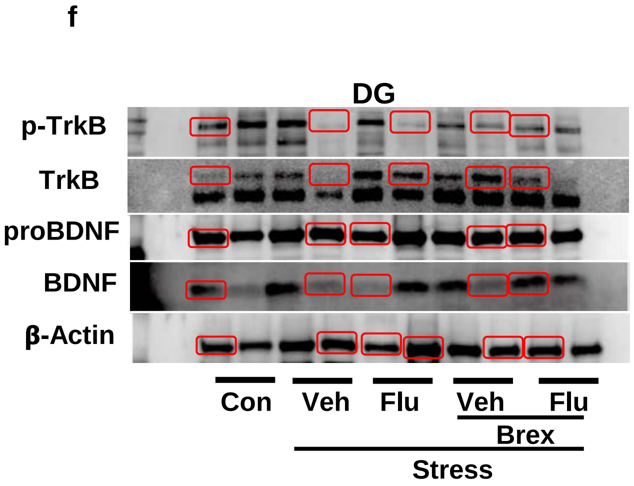

Supplemental Figure 4

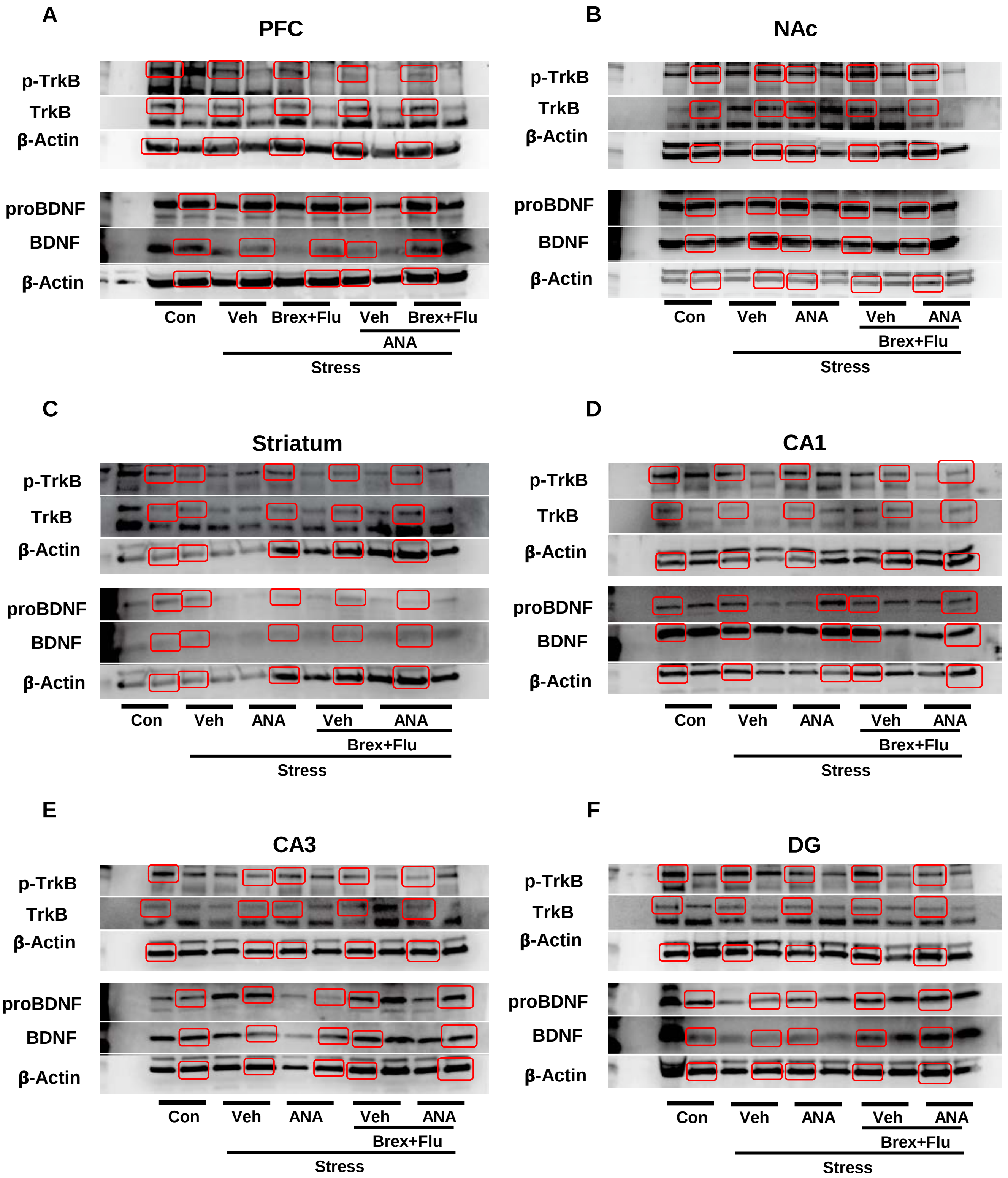

Supplement: Supplementary Information [file srep39209-s1.pdf]
